# Wound Healing in Streptozotocin-Induced Diabetic Rats Using Atmospheric-Pressure Argon Plasma Jet

**DOI:** 10.1038/s41598-018-30597-1

**Published:** 2018-08-15

**Authors:** Kuang-Yao Cheng, Zhi-Hua Lin, Yu-Pin Cheng, Hsien-Yi Chiu, Nai-Lun Yeh, Tung-Kung Wu, Jong-Shinn Wu

**Affiliations:** 1 Department of Mechanical Engineering, National Chiao Tung University, 1001 Ta-Hsueh Road, Hsinchu, 300 Taiwan; 2 Department of Biological Science and Technology, National Chiao Tung University, 75 Bo-Ai Street, Hsinchu, 300 Taiwan; 3 Department of Dermatology, Cathay General Hospital, 280 Renai Road Section 4, Taipei, 106 Taiwan; 4 Department of Dermatology, National Taiwan University Hospital Hsinchu Branch, 25 Jingguo Road Section 1 Lane 442, Hsinchu, 300 Taiwan; 5 Department of Family Medicine, National Taiwan University Hospital Hsinchu Branch, 25 Jingguo Road Section 1 Lane 442, Hsinchu, 300 Taiwan

## Abstract

In this study, we used an argon-based round atmospheric-pressure plasma jet (APPJ) for enhancing wound healing in streptozotocin (STZ) induced diabetic rats. The APPJ was characterized by optical emission spectroscopy. We induced Type 1 and Type 2 diabetes in rats with different amounts of STZ combined with normal and high-fat diets, respectively. The wound area ratio of all the plasma-treated normal and diabetic groups was greatly reduced (up to 30%) compared with that of the untreated groups during healing. Histological analysis revealed faster re-epithelialization, collagen deposition, less inflammation, and a complete skin structure in the plasma-treated groups was found as compared with the untreated control groups. In addition, the new blood vessels of plasma-treated tissues decreased more than untreated tissues in the middle (Day 14) and late (Day 21) stages of wound healing. The plasma-treated wounds demonstrated more transforming growth factor beta (TGF-β) expression in the early stage (Day 7), whereas they decreased in the middle and late stages of wound healing. The levels of superoxide dismutase (SOD), glutathione peroxidase (GPx), and catalase (CAT) increased after plasma treatment. In addition, plasma-treated water had a higher concentration of hydrogen peroxide, nitrite and nitrate when the plasma treatment time was longer. In summary, the proposed argon APPJ based on the current study could be a potential tool for treating diabetic wounds.

## Introduction

Diabetes mellitus is a metabolic disease and a common factor that results in chronic wounds due to abnormal blood glucose accumulation and causes neuropathy and arterial damage, affecting various tissues and organs^1^. Millions of people are suffering from it and consumed large number of medical costs. Generally, diabetes can be classified into two types: Type 1 and Type 2^2,3^. Type 1 diabetes is an autoimmune disease in which the β cells in the pancreas do not produce sufficient insulin, which is a hormone that helps maintain a proper balance of blood glucose for energy. Type 2 diabetes mellitus is a complex metabolic disease. The interaction between genetic and environmental factors results in a progressive disorder with variable degrees of insulin resistance and β-cell dysfunction. Obesity is a major contributor to the development of insulin resistance and impaired glucose tolerance^4,5^. Under these circumstances, intense inflammation due to neutrophil infiltration may contribute to delayed diabetic wound healing^6^. The high glucose environment also affects the interactions of growth factors such as insulin-like growth factors and vascular endothelial growth factors, thus resulting in poor re-epithelialization or angiogenesis^7–9^. Poor circulation, limited nutrients, and inflammation usually cause infections such as diabetic foot ulcer. Although antibiotics could be used to treat diabetic wounds, drug resistance is a common serious issue. Therefore, it is important to develop therapies from animal model to clinical trial to overcome those symptoms and shorten the diabetic wound healing process^10^.

The human body has different ways of protecting against potential threats from the environment. The skin is an effective barrier that protects the muscles, bones, and other organs and functions to maintain body fluid homeostasis^11^. A breach in the skin surface may cause serious disabilities or even death. Under normal condition, the healing process of small acute wounds takes only several days to weeks even without medical treatment. Generally, the repair of cutaneous wounds involves four phases: hemostasis, inflammation, proliferation, and remodeling^12,13^. Chronic wounds result from the failure of the normal wound healing process^14^. Any delay during the four wound healing phases could be defined as chronic wound healing. The formation of chronic wounds is complex and can be caused by ischemia, diabetes mellitus, and pressure. Improper treatment of the chronic wounds would lead to highly detrimental impact to human health. Thus, how to speed up and, thus, shorten the chronic wound healing process is a critical issue in modern dermatology.

Wound healing is dynamic involving the communication of cells, growth factors, and cytokines. For example, macrophages have various functions in the inflammation and proliferation phase, including the activation of inflammation, ECM synthesis, and supporting fibroblast proliferation^15–18^. Indeed, the cell-cell interaction is very complicated, and cell signaling requires ROS/RNS^19–21^. ROS/RNS not only inactivate bacteria but also react relatively quickly with proteins, cells, tissues, and even biological fluid. Hydrogen peroxide (H_2_O_2_) is a well-known ROS that participates in blood coagulation and wound contraction. It is also known for being the second messenger of tissue growth factor, platelet-derived growth factor, and vascular endothelial growth factor production. Owing to its high peroxidation activity, H_2_O_2_ is effective for bacteria inactivation. In addition, nitric oxide (NO) is another key species in wound healing for angiogenesis, inflammation, and tissue remodeling^22^. NO inhibits vascular smooth muscle contraction and growth, prevents platelet aggregation, and maintains vessel homeostasis. In addition, it can stimulate endothelial cell proliferation and prevent cell apoptosis, thus promoting the formation of new blood vessels in angiogenesis.

Recently, the application of non-thermal atmospheric-pressure plasma jet (APPJ hereafter) in the biomedical field has become increasingly popular^23–25^. It can be used not only in tissue engineering, surface modification, and sterilization but also in directly treating living cells or tissues such as skin^26–28^. The results revealed the upregulated expression of wound healing related factors including VEGF, FGF, HBEGF, and IL-6, which are responsible for angiogenesis or proliferation, after 120 or 180 second plasma treatment^28^. Some promising cases of treatment of chronic wounds in dogs and cats were presented which healed using kINPen in combination with both polihexanide and octenidine within 3 to 24 weeks^29,30^. In addition, some studies presented analysis of hundreds of MicroPlaSter plasma device treatments in tens of patients’ wounds with a highly significant reduction of bacterial load in infected wounds regardless of the type of bacteria and no side-effects occurred^31,32^. Plasma medicine focuses on enhancing the combination of reactive oxygen/nitrogen species (ROS/RNS), UV, ozone, and charged particles to cure various illnesses or pathological conditions^27,33^. Some studies have reported that the use of various gases, including helium-, argon-, and nitrogen-based APPJ, can improve the healing of different types of wounds^34,35^. Wound kinetics and histological results have demonstrated that burn or excisional wounds can be cured faster with plasma treatment^36–38^. In other words, the wound healing process can be promoted by an increase in ROS/RNS, which include H_2_O_2_, OH radicals, ozone, and NO generated in the plasma^39,40^. In our previous study, we have found that our in-house developed argon APPJ is ROS-rich and effective for surface modification and sterilization^10^. In addition, the safety test, based on the histology results, indicated that the plasma jet did not cause any thermal damage to the skin after continuous plasma jet treatment for slightly less than 10 minutes that is much longer than the treatment time planned in the current study^41^. In this study, we evaluated the efficacy of our self-developed argon APPJ in the wound healing of diabetic rats. We hope to use this argon APPJ with fixed parameters^10^ to improve diabetic wound healing and understand more detailed underlying mechanism.

## Experimental Methods

### APPJ device and instrumentation

**Figure 1:**
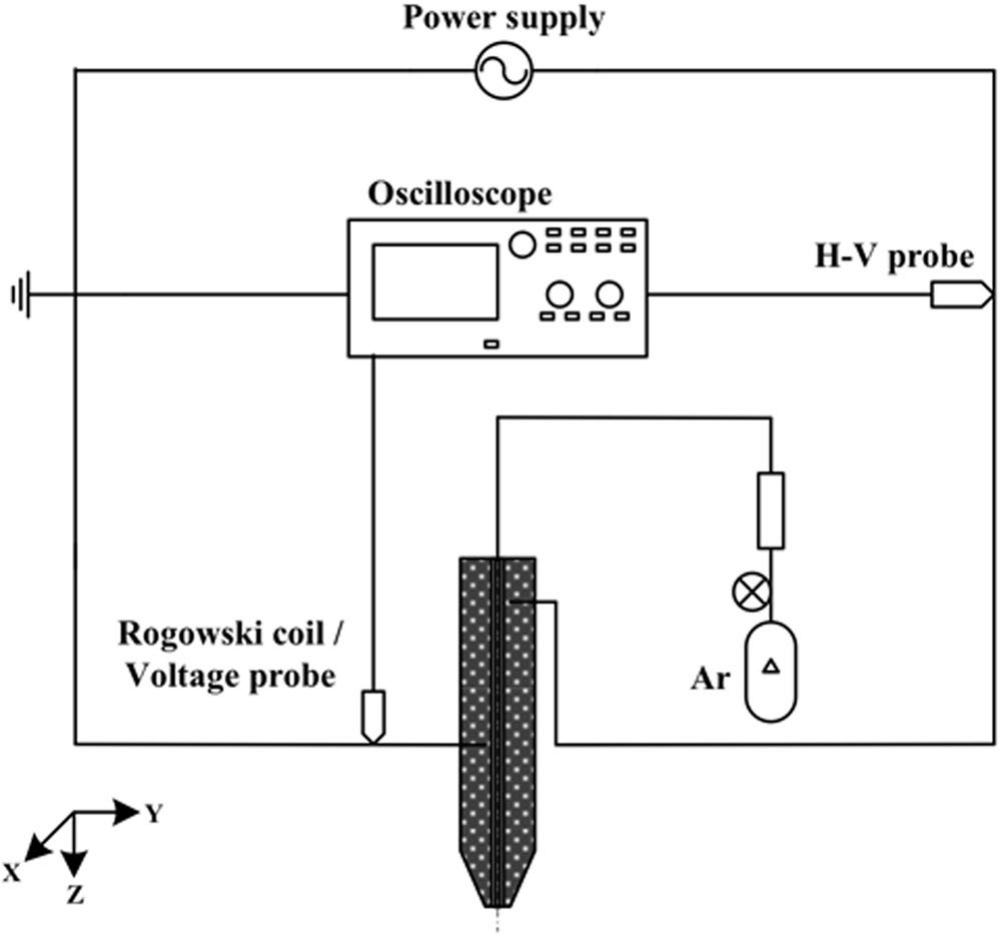
Schematic diagram of an argon round APPJ device with electrical measurement system.

Figure 1 shows the schematic diagram of an argon-based round APPJ device^21^ with electrical measurement instruments. The APPJ device consists of an in-house sinusoidal high-voltage compact power supply, a jet device with electrodes, and a gas bottle. We have determined the best experimental conditions of power control for plasma ignition and fixed the frequency at 20 kHz. Powered and grounded electrodes were made of a copper tube and covered by a quartz tube as a dielectric layer. A power electrode with 20 mm in length was placed at 50 mm above the end of the quartz tube, around which a ground electrode with 35 mm in length was wrapped. In addition, inside the quartz tube, we inserted a 20-mm floating electrode between power and ground electrodes and was in direct contact with gas flow. We made the protective covering of the device with ABS material using a 3D printer. The major discharge gas was argon (99.99%) with impurities (oxygen and water at a level of tens of and several ppm, respectively). Argon gas was flown from the gas bottle at a flow rate of 5 slm through the quartz tube and exhausted to the ambient or exposed on the surface.

Optical emission spectroscopy (OES) was performed to analyze excited species using an optical emission spectrometer (Model HD4000; Ocean Optics) with a resolution of 1 nm. The optical fiber was connected from the silt adapter (OES side) to the collimating lens (measuring side) (Model 74-UV; Ocean Optics). This lens had a focal length of 10 mm, which was used to receive collimated light with a diameter of approximately 5 mm. Data were obtained and analyzed with the PlasusSpecLine spectroscopy software.

### Wound healing in an animal model

All experimental protocols were approved by Animal Ethics Committee of National Chiao Tung University. The experiment was designed following animal welfare guidelines and the three Rs principles. We used male Sprague-Dawley (SD) rats (8-week-old, 275–300 g) (BioLASCO Co., Taiwan) as diabetic wound healing animal models. The rats were kept in an air-conditioned room at a constant temperature of 22 ± 1 °C and 55 ± 5% humidity with a 12-h light/dark cycle, in accordance with the Guide for Care and Use of Laboratory Animals^42^. Each rat was individually caged to prevent biting and fighting with other rats. All rats had free access to water and standard laboratory chow.

#### Diabetic model protocol

Streptozotocin (STZ) was used to induce diabetes in rats. According to several studies^3,43,44^, different amounts of STZ injection combined with normal chow and a high-fat diet can simulate human Type 1 and Type 2 diabetes, respectively. The Type 1 diabetic model was induced by a single, relatively high dose (60 mg/kg) of STZ (dissolved in 0.1 M citrate buffer, pH 4) intraperitoneal injection with normal chow (12% calories as fat). Before STZ injection, the rats were fasted overnight but were given access to water to prevent dehydration. STZ was given the following morning with recovery diet^45–47^. For the Type 2 diabetic model, male SD rats were fed a high-fat diet (60% calories as fat, 58Y1, TestDiet) for 2 weeks. A single intraperitoneal injection of 30 mg/kg STZ was given with recovery high-fat diet during the experiment^48^.

Blood glucose and insulin were measured to confirm whether the Type 1 and Type 2 diabetic models were successfully established. A week after STZ injection, the blood glucose levels of Type 1 and Type 2 diabetic rats were measured using a glucometer (GD40; Fora Care Inc.). We made a small incision on the rat tail using a surgical blade and absorbed the blood with blood glucose test strips to monitor glucose levels. When glucose level was higher than 300 mg/dL, the diabetic model was confirmed to be established. Type 1 and Type 2 diabetes can be easily distinguished by blood glucose levels. This study is to investigate the plasma treatment efficacy under hyperglycemia diabetic conditions. It is also well known that the use of insulin affects the wound healing speed. During the experiments, we monitored the glucose level of rats on a daily basis. We injected insulin only when the level exceeded 500 mg/dl to prevent the rats from becoming too weak and may lead to sudden death.

Additionally, in order to address individual differences that may cause uncertainties in the models, the Rat Insulin ELISA Kit (Abbexa Ltd.) was used to detect and quantify insulin. Insulin concentration is another factor that can be used to differentiate different types of diabetes. We drew blood from the rat tail using a syringe with a 25 G needle. The serum was coagulated at room temperature for about 2 h. Subsequently, it was centrifuged at 2000 rpm for 20 min. Measurements were performed according to protocol, and the optical density (O.D.) was determined at an absorbance of 450 nm using a microplate reader^3^.

#### Incisional wound model

The rats were anesthetized with 3% isoflurane during surgery. The hairs on the back were shaved using an electric clipper after the rats were unconscious. Two full-thickness round wounds were created on the both sides of the dorsum with an 8-mm sterilized biopsy punch for the small wound and with clippers based on a 21-mm sterilized Taiwanese coin for the large wound. For small wound healing, the rats were randomly divided into six groups: (1) normal group, in which wounds healed spontaneously without treatment; (2) normal group with plasma treatment; (3) Type 1 diabetic group, in which Type 1 diabetes was induced and wounds healed spontaneously without treatment; (4) Type 1 diabetic group with plasma treatment; (5) Type 2 diabetic group, in which Type 2 diabetes was induced and wounds healed spontaneously without treatment; (6) Type 2 diabetic group with plasma treatment. Each group contained twelve and six rats in small and large wound analysis, respectively. The animal number of small wound case was more than the large wound case because we conducted histology and antioxidants measurement using the former. In small wound healing case, we divided the experiment into two to make sure the efficacy on two different batches of rats can be reproduced. Therefore, on Day21, the total number of the rats in each group was four, which definitely met the statistical requirements. In large wound healing case, we did not conduct histology analysis, so we have used six rats to calculate the wound area calculation of each group. Thus, we have made sure all the number of experiments was statistically meaningful. The plasma jet was fixed at 5 mm distance above the skin. The treatment time depends on different types of animal and wound size. The range of treatment time on mouse are about 30–300 seconds in other studies^35,39,49–51^. In this study, we have used the same argon APPJ as employed in our previous study on plasma jet treatment of acute wounds. We have found the treatment was effective using 90 and 240 seconds of treatment time for relative small and large size acute wounds, respectively^52^. For small diabetic wound healing, plasma jet treatment time was fixed at 120 s once per day from the day the wounds were made until they were healed at the end of the experiment. For large wound healing, we increased the plasma jet treatment time to 240 s for a fair comparison between large and small wounds. The treatment for large wounds was performed by slowly circling the wounds from outside to inside to make sure all wound areas were treated.

### Wound healing analysis

#### Wound kinetics and calculation

Wound kinetics were recorded using a CCD camera each day from the day we made the wounds (Day 0) until the wounds healed in each experiment. A ruler was placed beside the wounds as a scale bar for area calculation and size comparison under various conditions. We calculated the daily wound area using an image analysis software (ImageJ), in which the wound area ratio was defined as the ratio of the current wound area to the initial wound area, to quantify the wound healing process for evaluating healing efficacy.

#### Hematoxylin and eosin staining

Four tissue samples of each condition were collected and made into blank slides at specific day for the followup stain uses. The rats were euthanized by CO_2_ on Day 7, 14, and 21 post-wounding. The wounds and adjacent skin were removed by punch biopsy with a diameter of 8 mm to ensure that the entire injury region was included; the removed tissues were placed on filter paper and soaked in 10% formalin solution for tissue fixation. After 24 h of fixation, the skin tissues were gradually dehydrated and embedded in paraffin. Then, a microtome was used to cut the tissues into 5-µm-thick sections. Hematoxylin and eosin staining was performed according to the manufacturer’s protocol.

#### Masson’s trichrome staining

Masson’s trichrome stain is a type of histochemical stain used to detect connective tissue maturation. Red represents keratin and muscle fibers, blue represents collagen, light red or pink represents the cytoplasm, and dark brown or black represents the cell nuclei. The depth and organization of the blue color can differentiate the maturity of collagen fibers. After blank histological sections were made, Masson’s trichrome staining was performed according to the manufacturer’s protocol.

#### Immunohistochemical staining

Immunohistochemical (IHC) slides for neovascularization and transforming growth factor beta (TGF-β) were prepared. For neovascularization expression, the sections were incubated with rabbit anti-CD31 polyclonal antibody (Abcam) at a dilution of 1:100 in PBS and then incubated at room temperature for 1 h. The staining was performed with the Discovery XT automated IHC/ISH slide staining system (Ventana Medical System, Inc., Tucson) using the ultraView Universal DAB Detection Kit (Ventana Medical System, Inc., Tucson) according to the manufacturer’s instruction. For TGF-β staining, the sections were incubated with TGF-β1 antibody (Fitzgerald) at a dilution of 1:100 in PBS, followed by the same procedures as described previously.

In order to quantify the numbers of stained cells and blood vessels for each sample, 10 high-power field (HPF) views at 400x magnification on each section were randomly taken and the number of cells were counted. The levels of inflammation, TGF-β expression and angiogenesis were evaluated. We used ImageJ to obtain the ratio of blue color (hue: 155–175; saturation: 60–255; brightness: 0–255) area in each HPF to represent the level of collagen deposition. In addition, the level of re-epithelialization was calculated as follows: (length of newly formed epidermis layer/length of wound between wound edges) × 100%. All counting procedures were conducted separately by two pathologists.

### Detection of antioxidants and ROS/RNS

#### Measurement of antioxidants

Superoxide is very chemically active and has a very short life time, and thus it is almost impossible to measure *in vivo*. To determine the effect of superoxide after plasma treatment on wounds, instead we measured the increase of SOD level *ex vivo* to support it is possible that superoxide species is originally contained in the APPJ or is synthesized during plasma treatment. Similar to the sampling procedure of histology analysis, we took the skins of small wound case at three temporal points (Day7, Day14 and Day21) for the measurements of antioxidants. Four rats of each experimental condition on each specific day were euthanized and then a piece of the skin including the wound and nearby uninjured part was removed by scissors. Each piece of removed skin was further chopped into small pieces with a weight of ~50 mg for each small piece. In order to extract and quantify proteins, the tissue was first placed in cold PBS. Then, it was repeatedly washed with 1 × PBS and 0.16 mg/mL heparin to remove blood clots and other debris, which are unnecessary impurities that would interrupt the transparency of the supernatant. Subsequently, the tissue was transferred to a petri dish on ice and cut into small pieces with surgical scissors. Finally, the tissue pieces were placed in a pre-chilled Eppendorf tube. For superoxide dismutase (SOD) detection, 1 mL of cold 1× cell extraction buffer was added to the Eppendorf tube. Then, the tissue was homogenized with Pellet Pestles Cordless Motor (Z359971; Sigma) and centrifuged at 10,000 × *g* for 10 min at 4 °C to remove insoluble pieces. The supernatant was transferred to a fresh tube on ice. Finally, the sample was placed in a 96-well microplate and mixed with SOD reagent, and the absorbance at 450 nm was measured at room temperature. For glutathione peroxidase (GPx) measurement, we homogenized the tissue with a homogenizer (Microtube Pellet Pestle Rod with Motor) in cold 1× assay buffer containing 0.4 mM PMSF and 1% Triton X‐100. After the sample was fully pulverized, it was centrifuged at 10,000 × *g* for 20 min at 4 °C to remove insoluble materials. The supernatant was transferred to a fresh pre-chilled tube on ice. Finally, the sample was pipetted into the bottom of a 96-well microplate and mixed with GPx reagent, and the absorbance at 349 nm was measured. For catalase (CAT) quantification, the homogenization process was similar to that for glutathione peroxidase, and the supernatant of the sample was pipetted into the bottom of a 96-well microplate. Next, 40 μM of H_2_O_2_ was pipetted into each well, followed by incubation for 30 min at room temperature. Finally, the reaction cocktail was added to each well, the plate was incubated for 15 min, and the fluorescence intensity at 590 nm (excitation 570 nm) was measured.

#### Measurement of ROS/RNS

We used DI water instead of wound fluids because the wounds we observed were easily dried out and thus it is not easy to obtain enough fluids from wounds for further plasma treatment and species quantification. Indeed, the species in DI water is different from the wound fluids. However, the major species of wound fluids is water, which motivated us to do a test like this. Note the purpose of using DI water to mimic real liquids of the rats was to show the capability of generating H_2_O_2_ and NO_2_^−^ by the argon APPJ treatment for 5, 10, 15, 30, 60, 90, 120, 150, 180, 240, and 300 s at a fixed distance 5 mm above water surface. We used the Amplex Red Hydrogen Peroxide/Peroxide Assay Kit (Invitrogen) to quantify the concentration of H_2_O_2_ in plasma-treated deionized water (DI water). The kit was used with horseradish peroxidase (HRP) to detect H_2_O_2_. The samples were pipetted into individual wells of the microplate with Amplex Red stock solution and HRP stock solution and incubated at room temperature for 30 min. The reaction was performed in the dark, and the absorbance at 560 nm was measured. For the detection of NO production, nitrite (NO_2_^−^) and nitrate (NO_3_^−^) were the final stable product of NO that should both be measured. Therefore, we used the Nitrite/Nitrate Colorimetric Assay Kit (Cayman) to measure the total amount of NO_2_^−^ and NO_3_^−^ to quantify the trend of NO production. The treated samples were pipetted into individual wells of the microplate with reagents. After the color was developed, the absorbance at 540 nm was measured using a plate reader.

### Statistical analysis

Data are expressed as the mean ± standard deviation (SD). Statistical analyses were performed with one-way analysis of variance (ANOVA) followed by post-hoc t-test. The test was conducted for comparison of each experimental condition with and without plasma treatment in wound area calculation, score of histological staining, and measurement of antioxidant levels. A P value of less than 0.05 was considered statistically significant and indicated with an asterisk (*).

## Results and Discussion

### APPJ characterization

**Figure 2:**
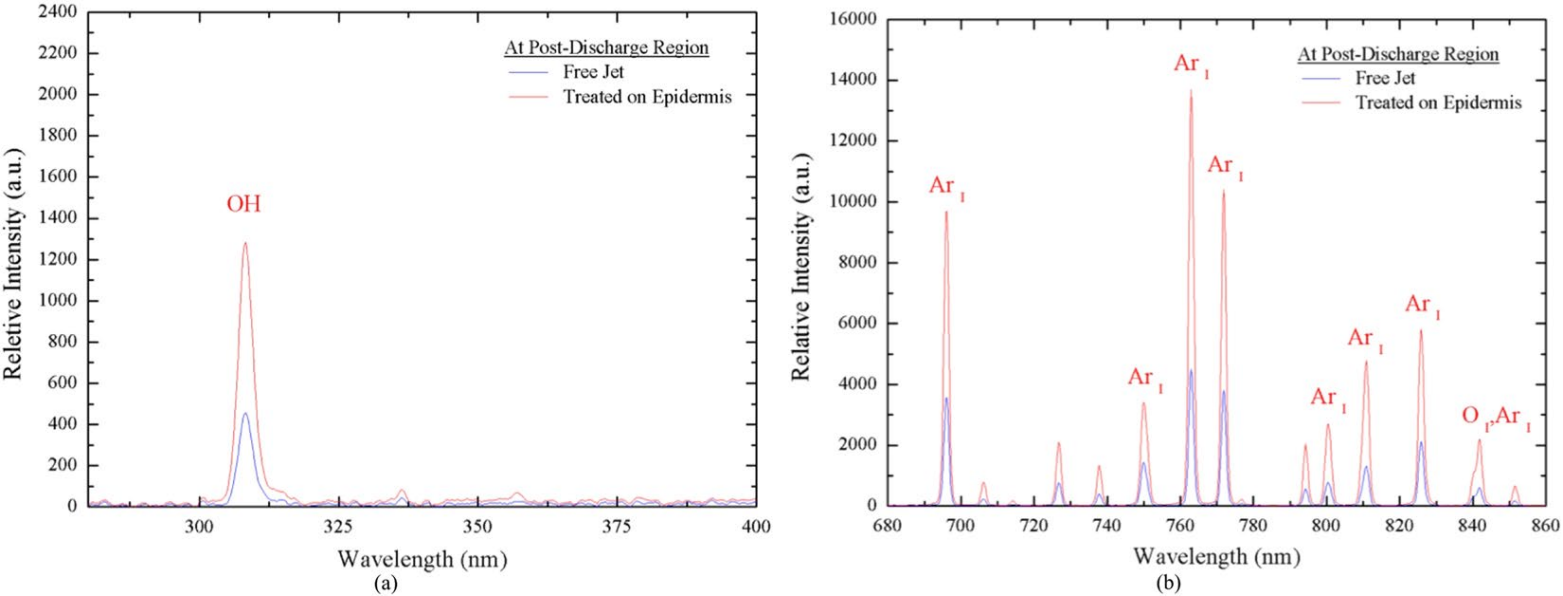
OES spectra analysis of argon plasma treated on epidermis and free jet (**a**) wavelength 280–400 nm; (**b**) wavelength 680–860 nm.

Figure 2 shows the OES spectral data of argon APPJ measured at x = 5 mm from the jet exit in a free jet and at an interface where the plasma jet interacts with the rat skin, which is the treatment distance to the wounds, in the UV range (280–400 nm) and visible range (680 nm–900 nm). The results revealed that the relative intensities of all measurements (OH, Ar, and O) were enhanced up to 2–3 times compared with those of the free jet when the plasma jet interacted with the epidermis of rats. It is possible that the epidermis acts as a floating electrode that interacts with the charged species of the plasma jet. Therefore, more excited species may be generated from the gas and humid ambient air due to the locally enhanced electric field at the interface^52^.

### Diabetic model

Table 1 shows the glucose, insulin concentrations and body weight of normal, Type 1 diabetic, and Type 2 diabetic rats. The method of diabetic model induction corresponded to the mechanisms of diabetes formation. The blood glucose concentrations of normal, Type 1 diabetic, and Type 2 diabetic rats were 128 ± 10, 483 ± 45, and 393 ± 30 mg/dl, respectively. The concentration in both diabetic groups was higher compared with that in the normal group, and Type 1 diabetes resulted in the highest blood glucose level. The insulin concentrations of normal, Type 1 diabetic, and Type 2 diabetic rats were 288 ± 127, 132 ± 35, and 215 ± 58 pmol/L, respectively. The insulin concentration in the diabetic groups was lower than that in the normal group, thus revealing the lack of insulin production. The low insulin concentration in the case of Type 1 diabetes may be attributed to severe β-cell damage. On the other hand, the insulin concentration in the case of Type 2 diabetes may be lower than or close to normal levels and depends on the level of β-cell destruction and insulin resistance, which is strongly correlated with high-fat diet-induced obesity. The body weight also demonstrated the effects of feeding different types of chow. The body weight of normal, Type 1 diabetic, and Type 2 diabetic rats were 408 ± 14, 402 ± 19, and 471 ± 25 g, respectively. In normal and Type 1 diabetic rats, due to normal chow feed, the body weight of both models were close. However, Type 2 rats showed a rapid weight gain caused by high-fat diet ingestion, which was one of the main factors of forming Type 2 diabetes. The combination of STZ-injection and high-fat diet indeed simulated the causes and indications of Type1 and Type 2 diabetes.

**Table 1:** Measurement of normal and diabetic characteristics (NC: normal chow; HFD: high-fat diet).

Condition	Normal	Type 1	Type 2
Method	NC	NC + 60 mg/kg STZ	HFD + 30 mg/kg STZ
Glucose Concentration (mg/dl)	128.5 ± 10.5	483 ± 45.82	393 ± 30.1
Insulin Concentration (pmol/L)	288 ± 27	132 ± 35	215 ± 58
Body Weight (g)	408 ± 14	402 ± 19	471 ± 25

### Small wound healing

**Figure 3:**
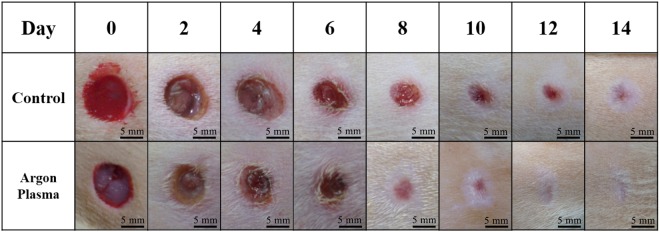
Wound kinetics of small wounds on various days during healing process with and without APPJ treatment. Scale bar: 5 mm.

Figure 3 shows the comparison of the healing of untreated and 120-s plasma-treated small normal wounds. The results revealed that in the early stage, which was around Day 2–6, the treated wound was smaller compared with the untreated control wound; the most distinct difference was around Day 7–10. Both treated and untreated wounds appeared to be completely closed on Day 14. These observations were quantified by the wound area ratio.

**Figure 4:**
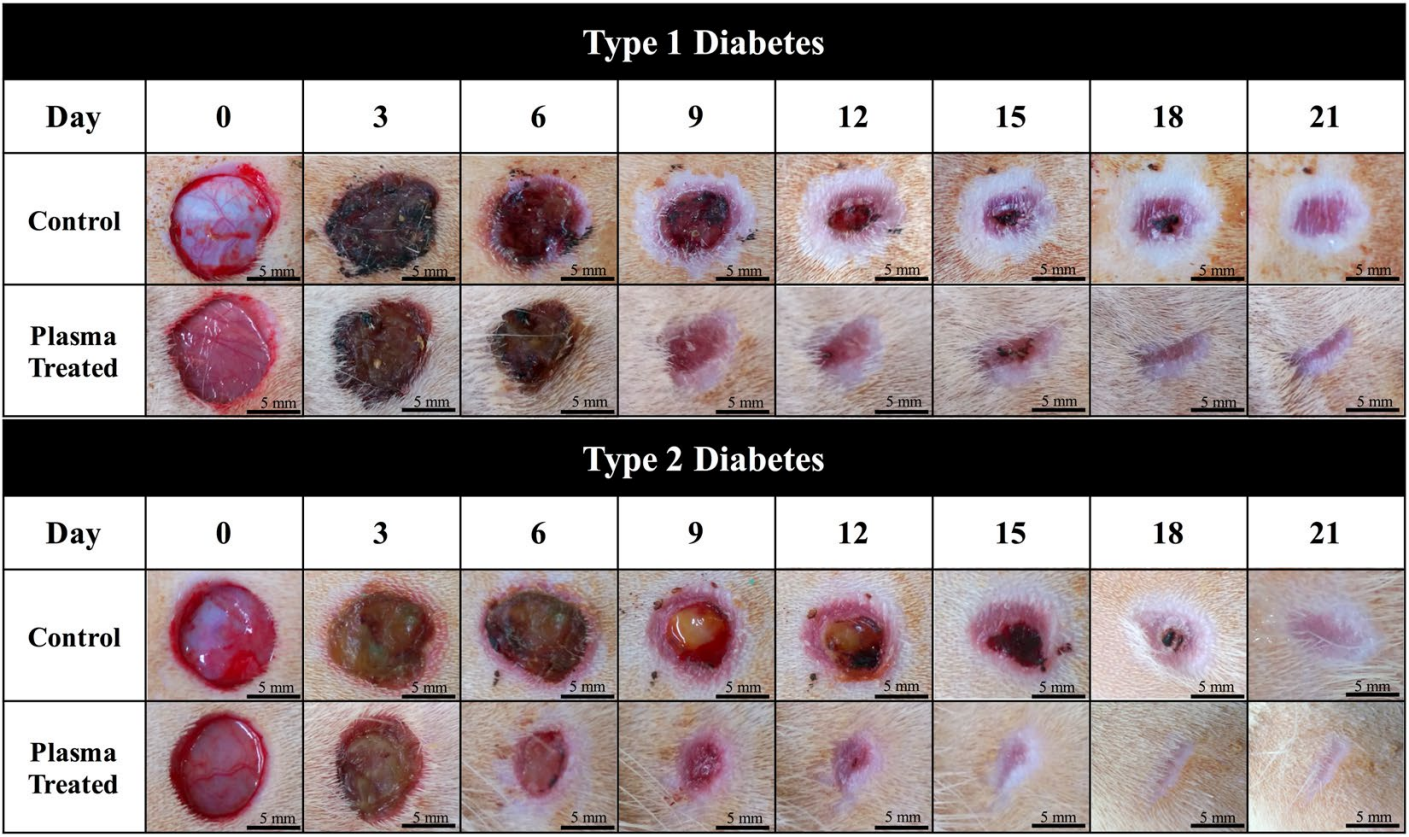
Wound kinetics of small wounds on various days during healing process for APPJ treated diabetic groups. Scale bar: 5 mm.

Figure 4 shows the wound kinetics of small wounds on various days during the wound healing process for the APPJ-treated (120 s) diabetic groups. In the plasma-treated Type 1 and Type 2 diabetic groups, healing was much faster than that in the control groups. In the case of Type 1 diabetes, the results showed that in the middle stage of the healing process (Day 9–12), remarkable differences in wound area and morphology can be observed. Re-epithelialization was faster in the plasma-treated group than in the control group. In the case of Type 2 diabetes, a faster recovery was also observed. Wound contraction was highly distinguishable around Day 6 and was greatly improved around Day 9–15. Similar to the case of Type 1 diabetes, re-epithelialization in the plasma-treated Type 2 diabetic group was faster than that in the untreated group.

**Figure 5:**
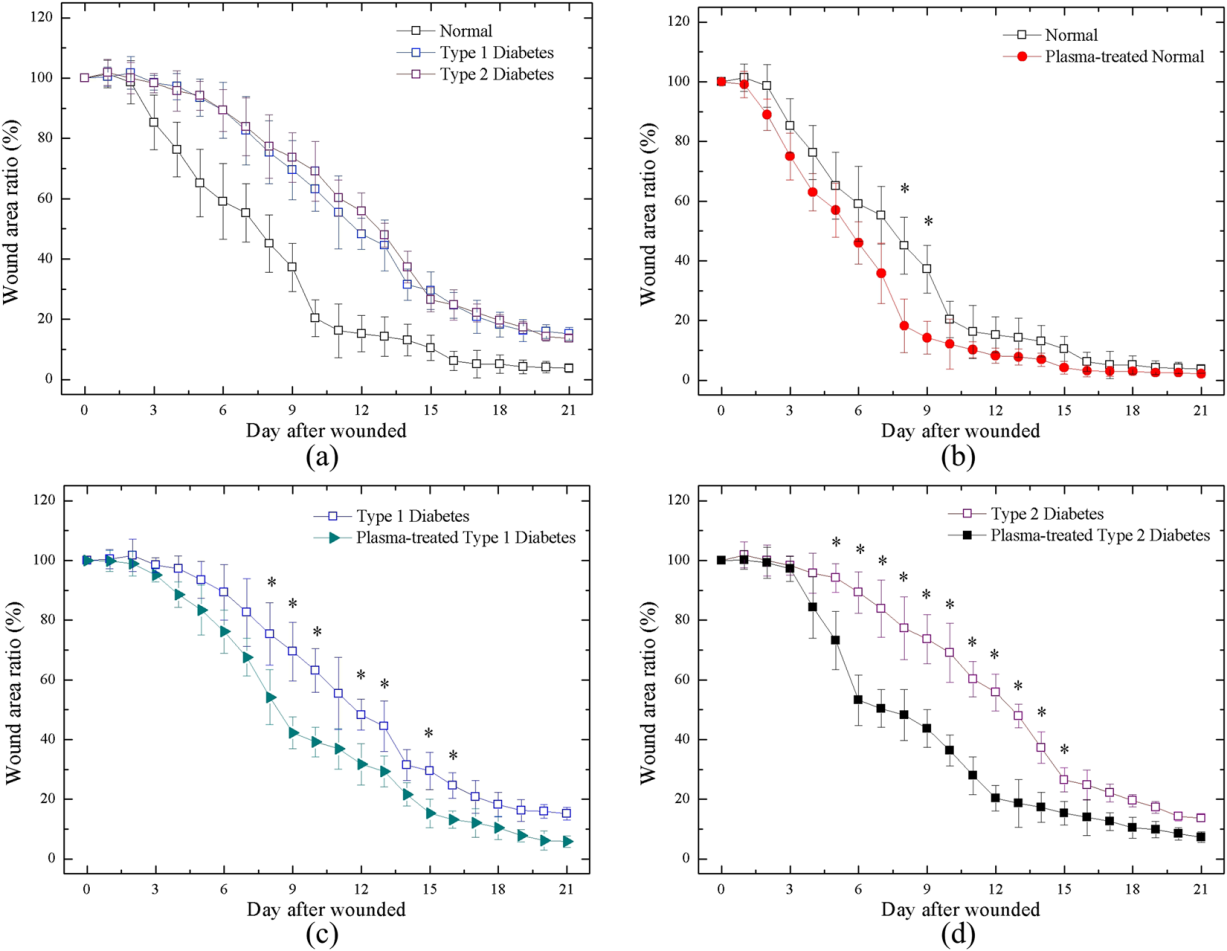
Wound area ratio during healing process of (**a**) all cases w/o treatment; (**b**) normal rats w and w/o plasma treatment; (**c**) Type 1 rats w and w/o plasma treatment; (**d**) Type 2 rat w and w/o plasma treatment. *P < 0.05 compared to control group.

Figure 5 shows the small wound area ratio of the untreated and plasma-treated groups during the wound healing process. We calculated the wound area ratio of each group using *ImageJ* software. We selected the threshold of the gray level to define the wound boundary between the wound and healthy areas, which can introduce some uncertainties in quantification. The calculation was performed up to Day 21 because of the slow healing speed of diabetic wounds. A comparison of the healing of the untreated normal, Type 1 diabetic, and Type 2 diabetic groups was performed as shown in Fig. 5(a). There was a considerable difference between the wounds of the untreated normal and diabetic groups. The healing patterns of the two diabetic groups were similar; however, their average contracting speed was much slower than that of the normal group. In early stage around Day 3, distinct differences can be observed and were consistent until the completion of healing. In the middle stage, the wound area ratio was reduced up to 40~50% between Day 9 and 12. As shown in Fig. 5(b), in the untreated and plasma-treated normal groups, the results revealed that the wounds of both groups were almost completely healed on Day 15; moreover, between Day 16 and 21, the wound area ratio was nearly similar and overlapped between the groups. Figure 5(c) shows the wound contraction of the plasma-treated and untreated Type 1 diabetic groups. Healing was promoted through plasma treatment, and the wound area ratio was decreased up to 30% between Day 7 and 10. Figure 5(d) shows the wound contraction of the Type 2 diabetic group, which revealed that plasma treatment was largely effective in improving healing. Healing was faster in the plasma-treated group than in the untreated group from Day 6 to 14. In addition, the wound area ratio was reduced to 40% on Day 6 and remained at around 30% from Day 7 to 13.

**Figure 6:**
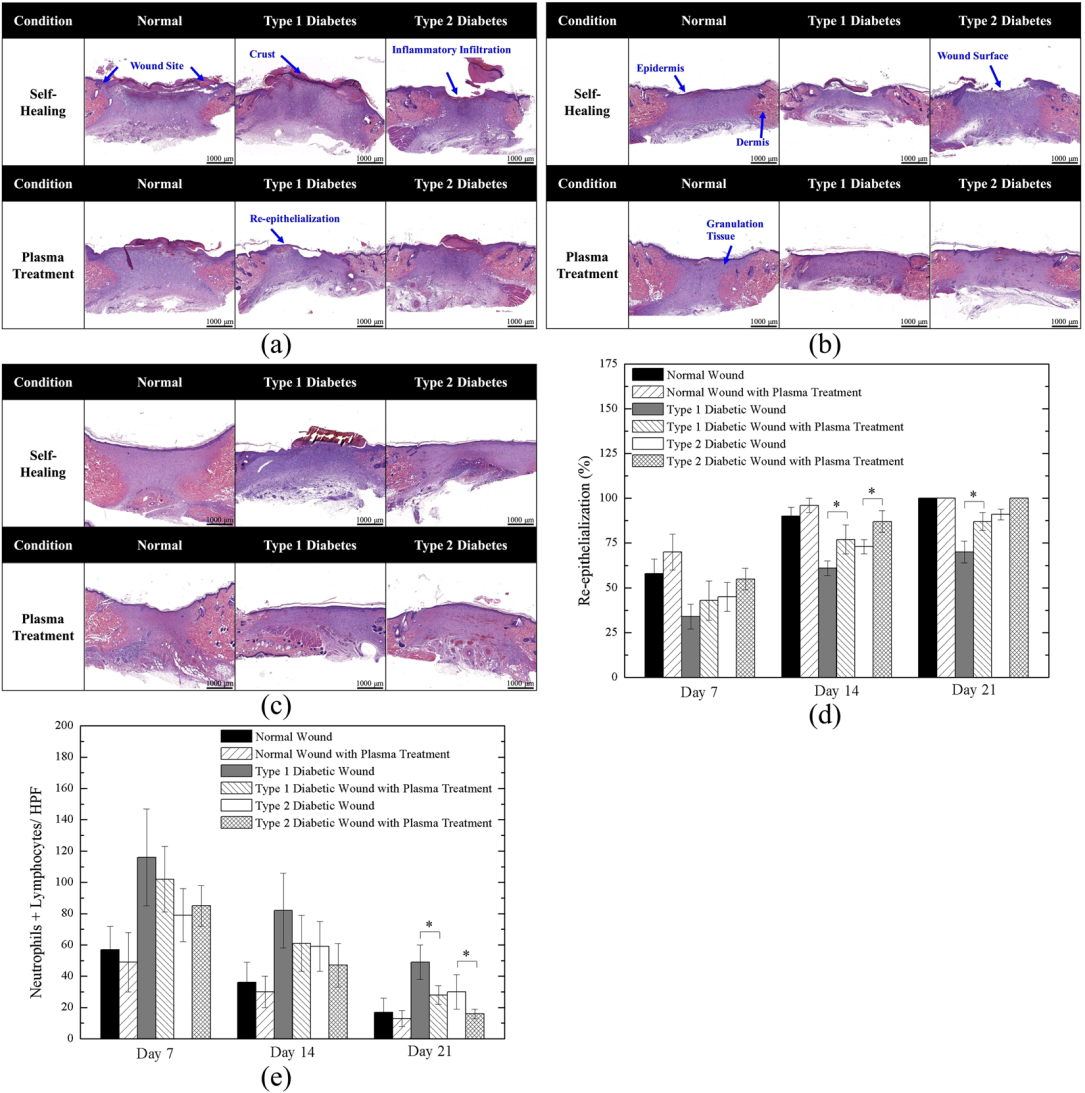
Hematoxylin and Eosin Staining of (**a**) Day 7; (**b**) Day 14; (**c**) Day 21 wounds; (**d**) quantitative re-epithelialization; and (**e**) quantitative inflammation. *P < 0.05 compared to the untreated group. (Scale bar: 1000 µm; HPF: 400x).

H&E staining results of Day 7, 14, and 21 wounds are shown in Fig. 6, corresponding to the early, middle, and final stage of wound healing, respectively. In Fig. 6(a), a clear wound border can be observed in all six cases. However, in most groups, the re-epithelialization process was occurring, notably extending to the wound surface except in the Type 1 diabetic group without plasma treatment, which showed unclear epithelium migration. Inflammation was observed in all non-treated groups; however, plasma treatment could decrease inflammatory effects. The structure of the epidermis and dermis layers can be distinguished as shown in Fig. 6(b). Nevertheless, the untreated diabetic groups showed poor keratinocyte migration. The untreated Type 1 and Type 2 diabetic groups had an exposed wound surface, which indicated that re-epithelialization in these groups was much slower than that in the plasma-treated diabetic and normal groups. In addition, the rate of granulation tissue maturation in the plasma-treated group with normal wounds was the highest, which corresponded to the results of wound kinetics. In the final stage of wound healing, as shown in Fig. 6(c), all wounds were almost completely healed. However, similar to the previous results, the healing of the untreated Type 1 diabetic group was delayed. Additionally, we found that the maturation level of the three plasma-treated groups was higher than that of the untreated groups, as indicated by the size of the purple area. Figure 6(d,e) show the quantitative re-epithelialization and inflammation, respectively. The plasma treated cases showed higher re-epithelialization level especially more pronounced in the diabetic wounds in the late stage. Both Type 1 and Type 2 diabetic wounds showed significant difference after plasma treatment on Day 14. It is worth noting that in the late stage (Day 21), which can also be observed in Fig. 6(c), the plasma-treated Type 1 diabetic wounds presented significant enhancement compared to untreated Type 1 wounds. Also, in the normal group, normal group with plasma treatment, and Type 2 diabetic group with plasma treatment reached 100% on Day 21. For quantitative inflammation as shown in Fig. 6(e), the plasma treated groups demonstrated lower inflammation levels in average, and only the plasma-treated Type 1 and Type 2 diabetic groups decreased significantly on Day 21. In addition, for the normal groups, including untreated and plasma-treated cases, the number of inflammatory cells were less than the diabetic groups because diabetes mellitus led to increase of inflammation^53^.

**Figure 7:**
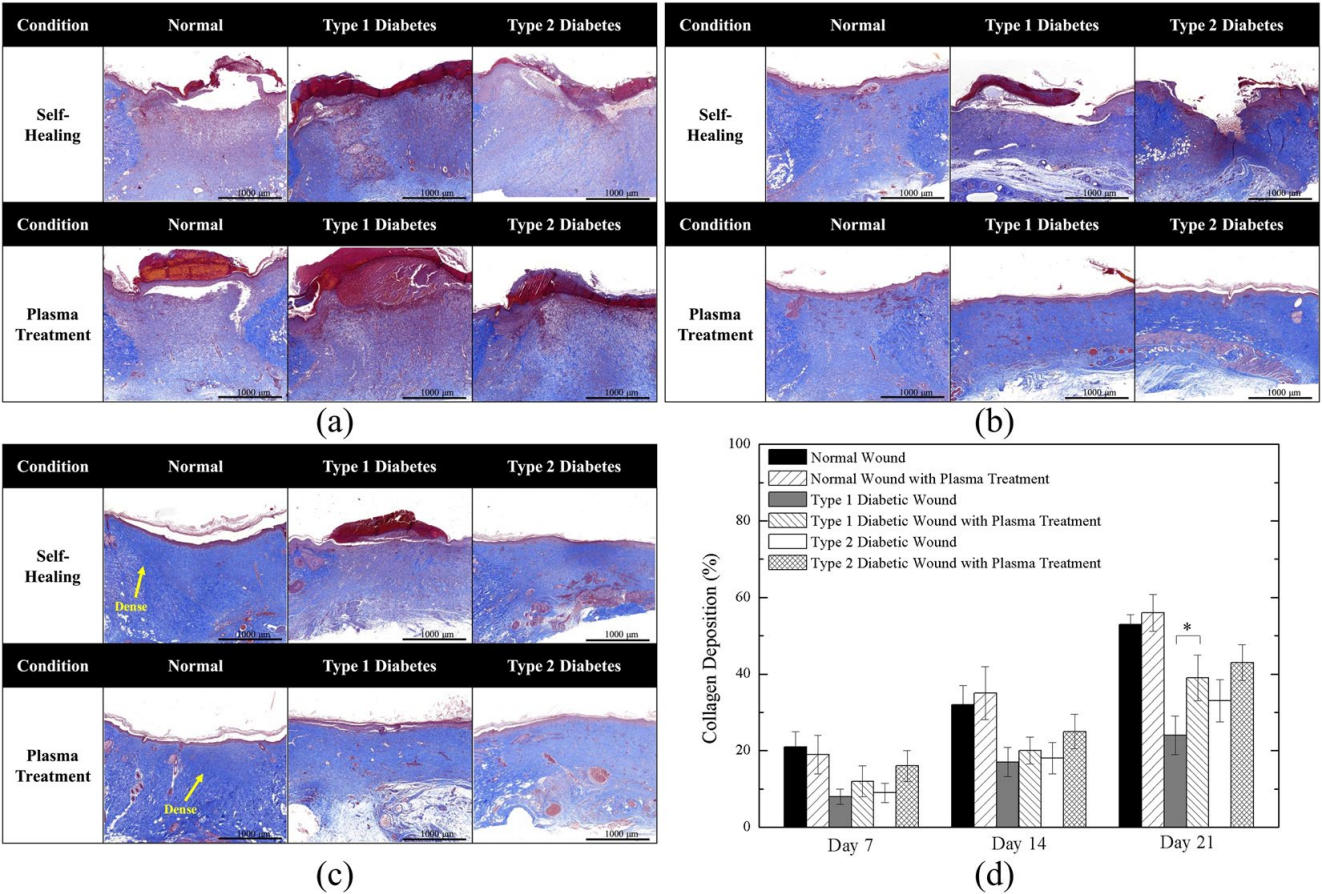
Masson’s Trichrome Staining of (**a**) Day 7; (**b**) Day 14; (**c**) Day 21 wounds; and (**d**) quantitative collagen deposition. *P < 0.05 compared to the untreated group. (Scale bar: 1000 µm).

Figure 7 shows the Masson’s trichrome staining of Day 7, 14, and 21 wounds. As shown in Fig. 7(a), in the early stage of the healing process, collagen formation on the wound site was rare. This may be because the wounds were just about to complete inflammation and begin proliferation in the early stage; thus, collagen deposition was minimal. In Fig. 7(b), noticeable color changes can be observed. The results demonstrated that normal wounds with and without plasma treatment had the highest level of collagen deposition. In both Type 1 and Type 2 diabetic cases, plasma-treated wounds had moderate collagen deposition compared with untreated wounds. Figure 7(c) shows the final stage of wound healing. The blue color of the normal wounds was the most intense, indicating highly mature collagen fibers. Plasma-treated Type 1 and Type 2 diabetic wounds showed moderate collagen deposition, whereas untreated Type 1 diabetic wounds still showed poor collagen deposition. Figure 7(d) shows the quantitative collagen deposition. The levels of plasma-treated and untreated normal groups were close in all stages and remained the highest among all cases. On the other hand, the plasma-treated Type 1 diabetic group significantly increased collagen deposition on Day 21.

**Figure 8:**
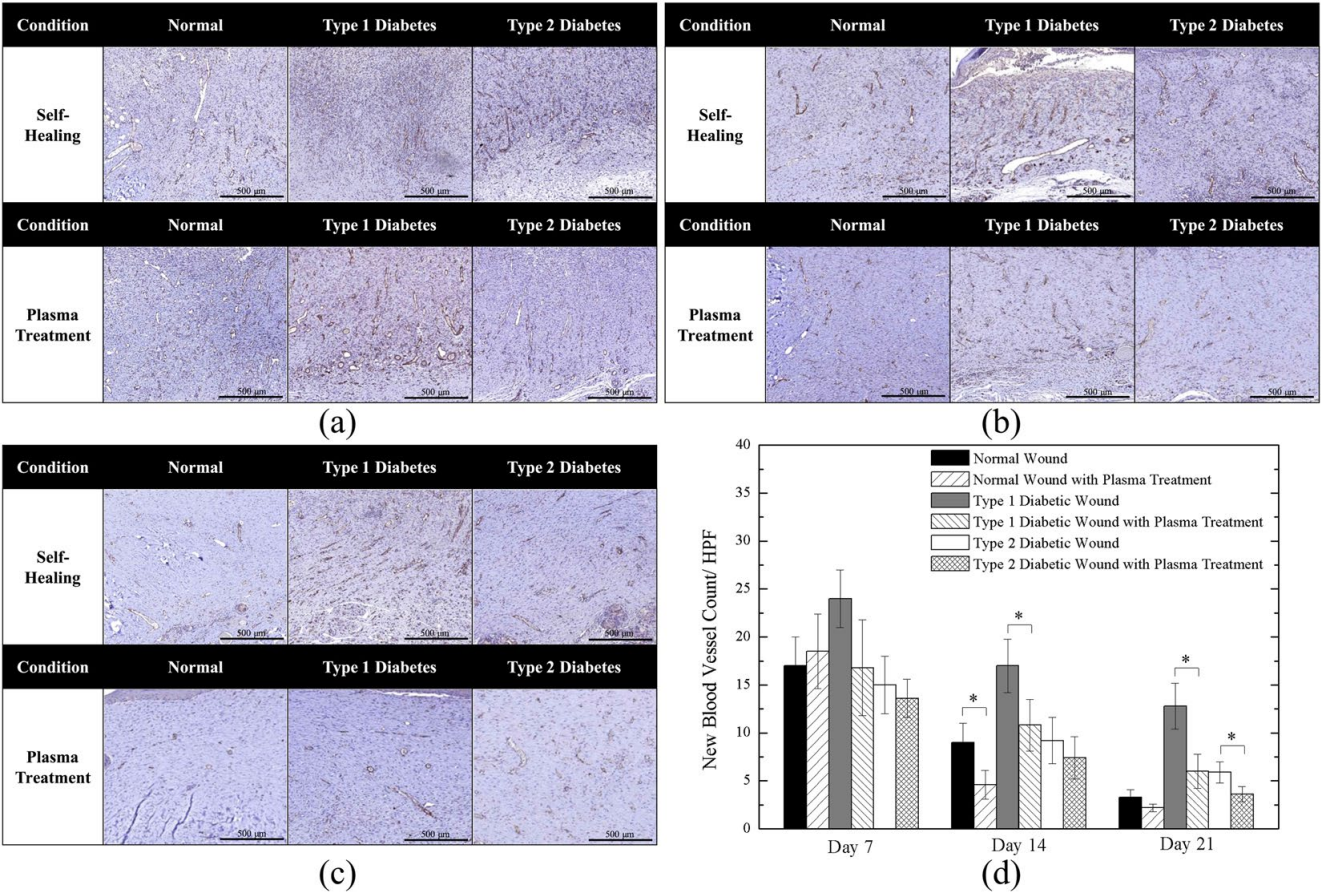
CD31 Staining of (**a**) Day 7; (**b**) Day 14; (**c**) Day 21 wounds; and (**d**) quantitative angiogenesis. *P < 0.05 compared to the untreated group. (Scale bar: 500 µm; HPF: 400x).

**Figure 9:**
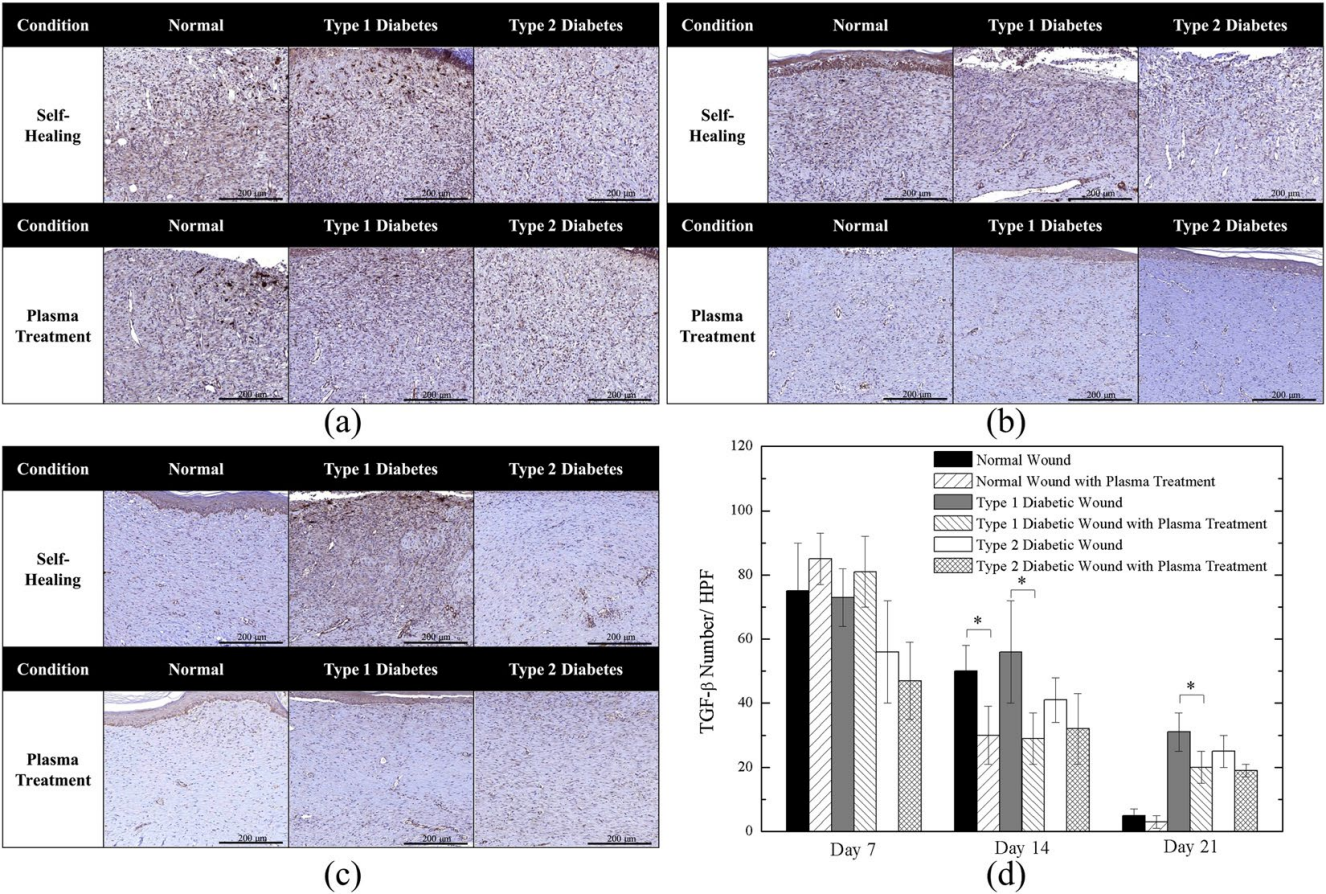
TGF-β Staining of (**a**) Day 7; (**b**) Day 14; (**c**) Day 21 wounds; and (**d**) quantitative TGF- β expression. *P < 0.05 compared to the untreated group. (Scale bar: 200 µm; HPF: 400x).

Figures 8 and 9 show the IHC staining of CD31 and TGF-β, respectively. Figure 8(a) shows the neovascularization in the early healing stage. All cases demonstrate existence of new vessels; furthermore, from the quantitative angiogenesis as shown in Fig. 8(d), the untreated Type 1 diabetic wound presented the highest number of new blood vessels. In Fig. 8(b), the amounts of new vessels of the untreated cases reduced on Day 14 and they were further reduced by the plasma treatment which is quantitatively shown in Fig. 8(d). The above findings were in consistent with previous studies^54,55^ which stated that CD31 expression decreases during the later stage of wound healing when the number of blood vessels started to decline and the density of blood vessels returned to the level of uninjured skin. Figure 8(c) shows the wound on Day 21, in which new vessels were not present, except in the case of the untreated Type 1 diabetic wound and it is also shown quantitatively in Fig. 8(d). In addition, Fig. 8(d) shows that both Type 1 and Type 2 diabetic wounds with plasma treatment exhibited significantly reduced new-formed vessels on Day 21. The delayed healing process resulted in delayed vessel formation, and as a result normal wound groups presented the relatively low number of new vessels in middle and late stages.

Figure 9(a) shows the staining of TGF-β on Day 7. In this stage, inflammation and cell proliferation led to TGF-β secretion. The results revealed that extracellular TGF-β was highly expressed (brown color) in all cases (the dark brown color of the crust in some cases should be ignored), which are also shown in Fig. 9(d). The quantitative analysis in Fig. 9(d) shows that the plasma-treated normal and Type 1 diabetic wound might increase TGF-β number in the early stage (Day 7). Figure 9(b) demonstrates wound maturation progressed with decreasing TGF-β levels in the middle stage. Noticeably, as Fig. 9(d) shows, the Type 1 diabetic untreated group remained relatively high level of TGF-β secretion as compared to the case of plasma treatment on Day 14. In the late stage, as shown in Fig. 9(c), TGF-β expressions were rarely observed except for the untreated healed Type 1 diabetic tissue that might result from the prolonged and delayed wound healing process. On the other hand, after plasma treatment, the Type 1 diabetic wound decreased significantly. It is worth noting that both untreated and plasma-treated groups of normal wounds showed very few TGF-β on Day 21 because the wounds healed more completely than other groups.

The overall results based on the above various staining observations indicated that with plasma treatment may increase the amount of free radicals involved in cell signaling and affect some pathways, thus accelerating the healing process of normal and diabetic wounds.

### Large wound healing

**Figure 10:**
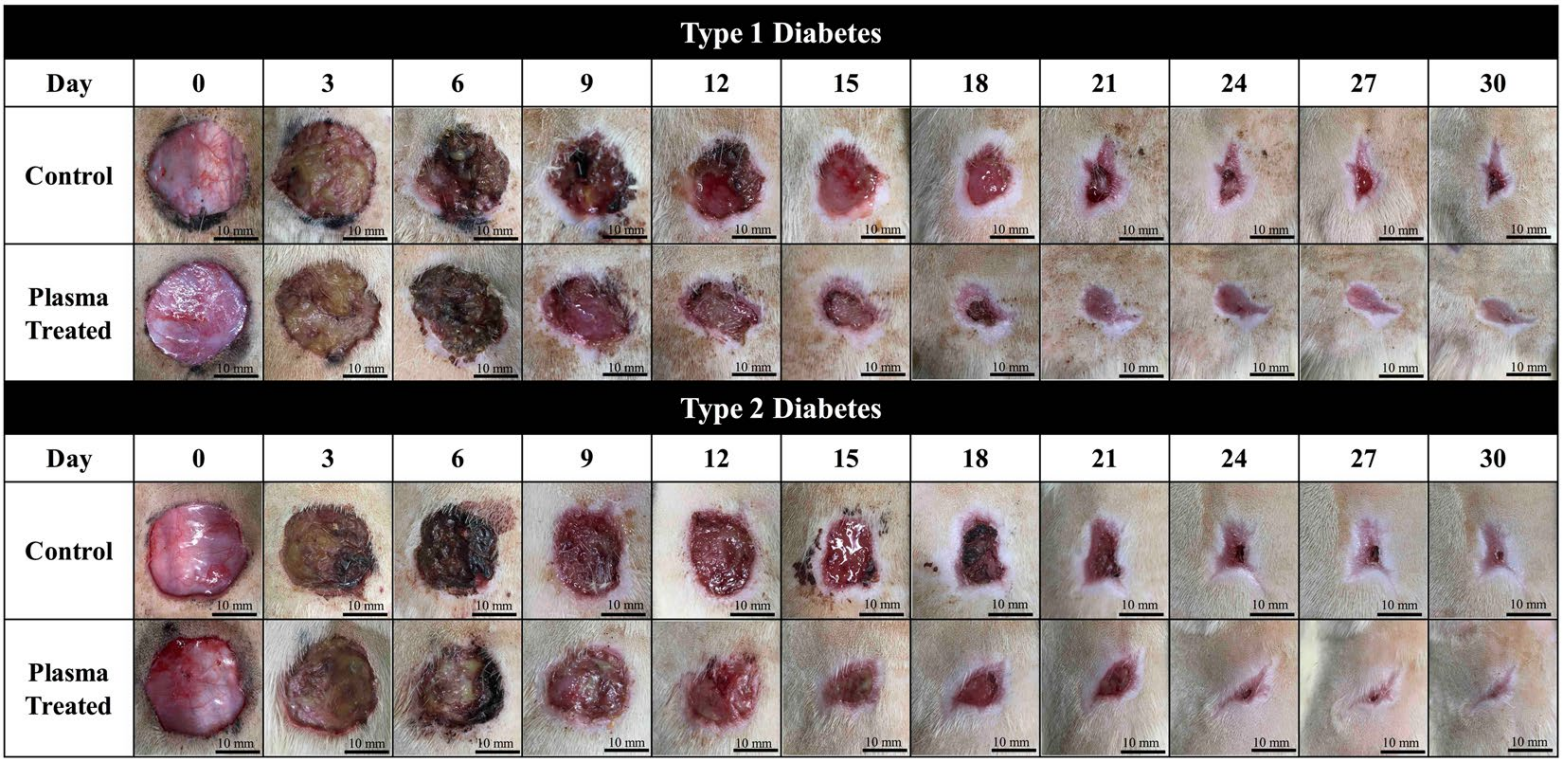
Wound kinetics of large wounds on various days during healing process for APPJ treated diabetic groups. Scale bar: 10 mm.

**Figure 11:**
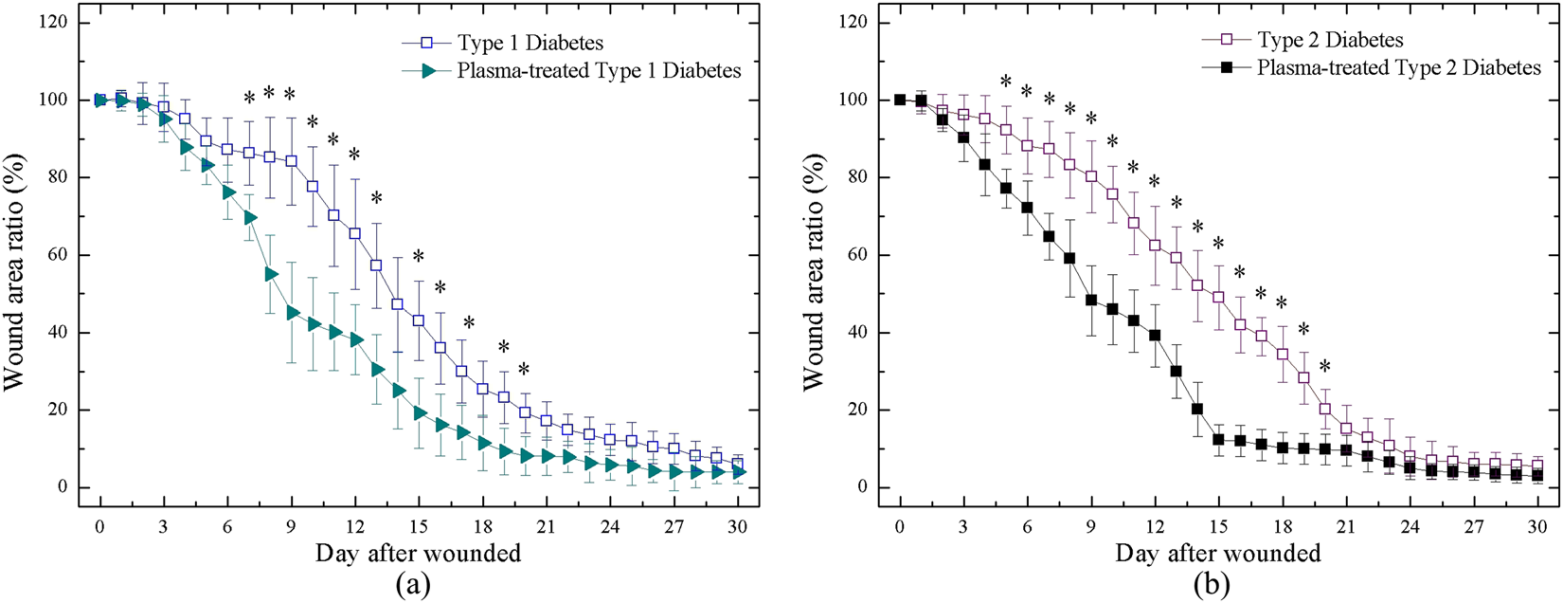
Wound area ratio during healing process of (**a**) Type 1 rat w and w/o plasma treatment; (**b**) Type 2 rat w and w/o plasma treatment. *P < 0.05 compared to control group.

Figure 10 shows the wound kinetics of large wounds on various days during the wound healing process for the APPJ-treated diabetic groups. Plasma treatment in both the Type 1 and Type 2 diabetic groups was observed to promote healing. There were distinct differences in the middle stage (around Day 9–15). Nevertheless, in the late stage, the wounds with plasma treatment is only slightly better than without plasma treatment. The wound healing time during maturation was longer than in other healing stages; thus, the healing speed of untreated and plasma-treated wounds ended up being similar. However, the faster wound healing in the initial and middle stages is very important for preventing the wound from serious bacterial infection during the course of healing. Figure 11 shows the large wound area ratio of the untreated and plasma-treated groups during the wound healing process. In the case of Type 1 diabetes, the wound area was substantially reduced up to 30~40% in the plasma-treated group compared with the untreated group, especially between Day 7 and 12; however, the healing speed was lower from Day 18 to 30. In the case of Type 2 diabetes, the reduction was maintained at a ratio of 20~30% from Day 7 to 18. This enhanced wound healing speed in this period is important for a wound healing process which reduces the risk of bacterial infection. Overall, in the late stage of the healing process, the wound area ratio with or without plasma treatment was almost similar and was consistent with previous results.

### Measurement of antioxidants

Antioxidants are molecules that catalyze ROS into less or unreactive species such as oxygen and water. SOD scavenges superoxide anion and converts to hydrogen peroxide, and then GPx and CAT catalyze hydrogen peroxide to oxygen and water^56^. Thus, the quantities of SOD, GPx and CAT may strongly correlate with the existence of superoxide anion, which is induced due to the radical generation by the plasma jet treatment to the tissues. We will present the corresponding measured data next in turn.

**Figure 12:**
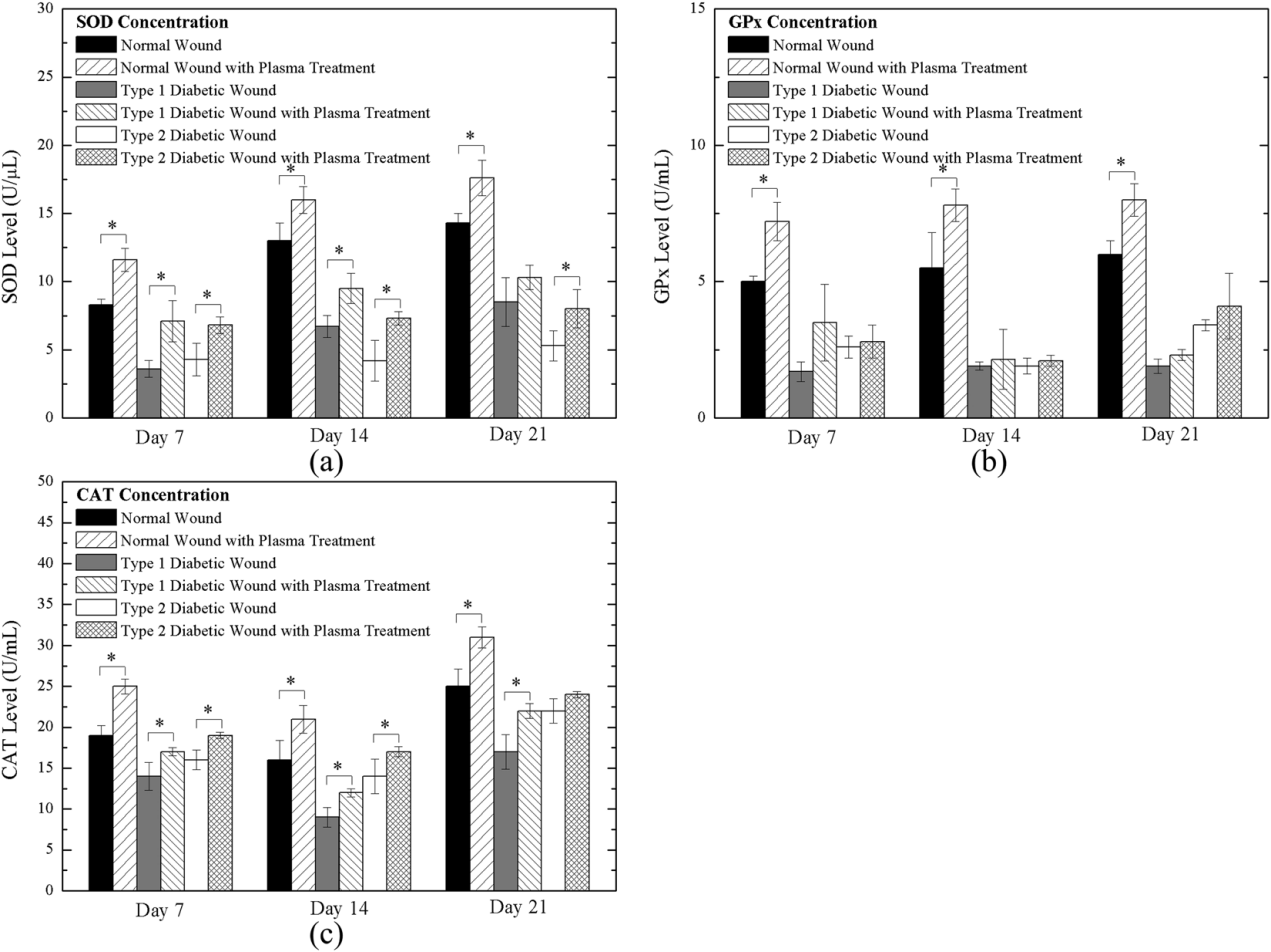
Antioxidant level of Day 7, Day 14 and Day 21 wound of (**a**) SOD; (**b**) GPx; and (**c**) CAT. *P < 0.05 compared to the untreated group.

Figure 12(a) shows the concentration of SOD in the tissues of the normal, Type 1 diabetic, and Type 2 diabetic rats (without and with daily plasma treatment for 120 s) on Day 7, 14, and 21 during the healing process. In all normal, Type 1 diabetic, and Type 2 diabetic rats with plasma treatment, SOD levels obviously increased. The SOD level in normal rats was higher than that in diabetic rats because of higher normal antioxidant activity. In the late stage, average SOD levels were higher than in the early and middle stage in normal and Type 1 diabetic rats. It was assumed that the plasma jet contained superoxide anion and increase the level of SOD after treatment. Figure 12(b,c) show the GPx and CAT levels of normal, Type 1 diabetic, and Type 2 diabetic rats (without and with daily plasma treatment for 120 s) on Day 7, 14, and 21 during the healing process. Both GPx and CAT levels were increased after daily plasma treatments. The results demonstrated that free radicals participated in the wound healing process due to the increase in antioxidants. The lower antioxidant levels of diabetic rats may result from oxidative stress in a high-glucose environment^57^. In diabetes, constant hyperglycemia can increase the production of free radicals via the oxidation of glucose and the glycation of non-enzymatic proteins, and a decrease in SOD, GPx, and CAT has been reported^58,59^.

Free radicals including superoxide anion and H_2_O_2_ participate in the wound healing process, increasing antioxidant levels. The increased levels of antioxidants also lead to a decrease in excess ROS to reduce oxidative stress, thus promoting wound healing^60^. On the other hand, exogenous ROS/RNS of the plasma jet could improve re-epithelialization, granulation formation, and angiogenesis according to histological results.

### Measurement of ROS/RNS

**Figure 13:**
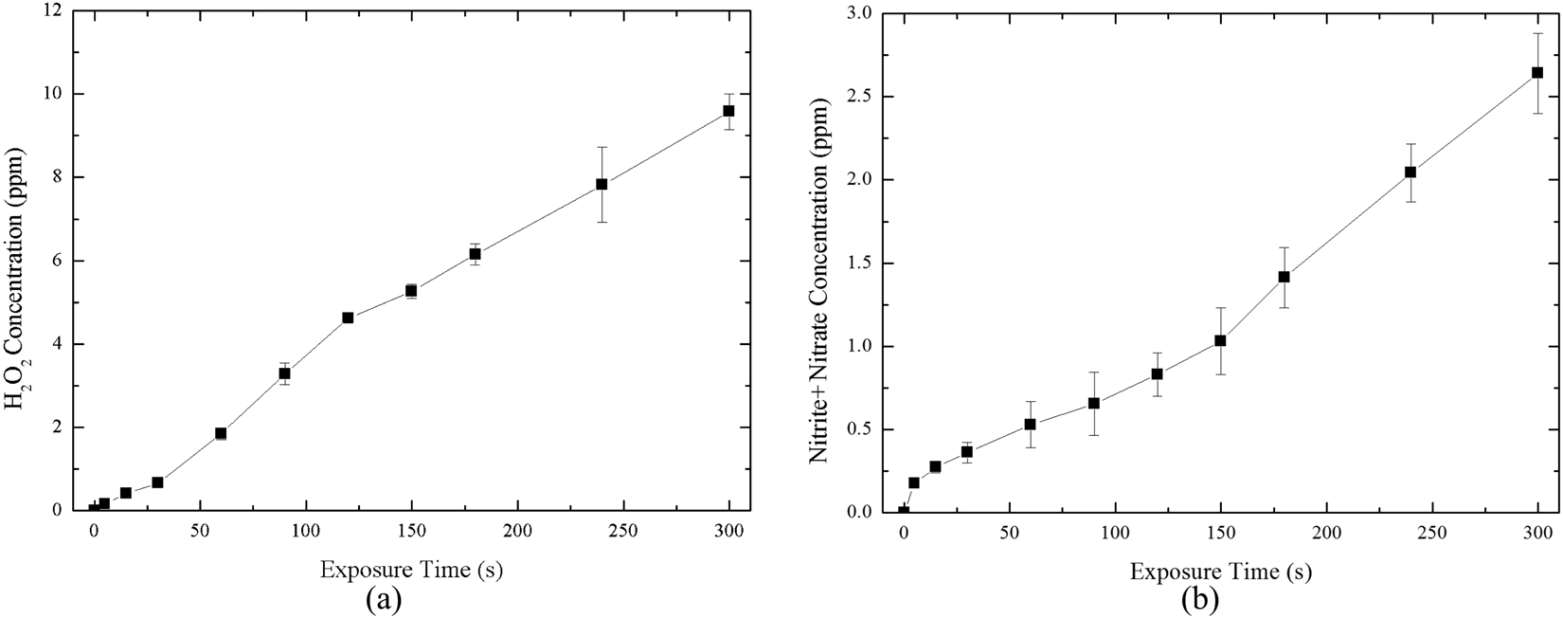
Measurement of (**a**) H_2_O_2_ and (**b**) nitrite and nitrate concentration in plasma-exposed DI-water on various treating time.

Figure 13(a) shows the H_2_O_2_ quantification of plasma-treated DI water for various treatment times. The concentration was about 4.6 ppm and 7.8 ppm at a plasma treatment time of 120 s and 240 s, respectively. An increase in treatment time was demonstrated to correspond to an increase in H_2_O_2_ concentration. Figure 13(b) shows the NO_2_^−^ plus NO_3_^−^ quantification of plasma-treated DI water with various treatment times. The results indicate that NO production increases with increasing treatment time. For example, the concentration was increased to 0.17 ppm in a short time, and the concentration was 0.83 ppm and 2.04 ppm at a plasma treatment time of 120 s and 240 s, respectively. Since the tissue contains body fluids, according to the results, the free radicals in the plasma could react with these liquids, which indicated the involvement of cell signaling and antioxidation.

The overall results based on the above various staining observations indicated that plasma treatment may increase the amount of free radicals involved in cell signaling and affect some pathways, thus accelerating the healing process of normal and diabetic wounds. Superoxide has been known to have high activity and a very short life time^61^. It is possible that superoxide species is either originally contained in the APPJ or is synthesized during plasma treatment^62,63^. After exposing the wounds to the plasma jet, plasma-generated superoxide anion and hydrogen peroxide may directly attack invading bacteria and other pathogens^56,64^. The excess ROS, which gave rise to the activity of SOD (Fig. 12(a)), might stimulate angiogenesis and re-epithelialization along with NO to promote healing to next stage^65^. It was proved by the increase of GPx and CAT level (Fig. 12(b,c)) that H_2_O_2_ affected cells after daily plasma treatment. Salehi *et al*.^34^ observed that a high level of oxidative stress (antioxidants increased after plasma treatment) led to destruction of bacteria accumulation and stimulation of reepithelization. During wound healing process, angiogenesis is induced by tissue hypoxia and ROS which activate macrophages, fibroblasts, endothelial cells, and keratinocytes (Figs 6 and 7) to produce VEGF. It was reported that exogenous ROS stimulate the induction of VEGF expression in various cell types, such as endothelial cells (Fig. 8), smooth muscle cells, and macrophages^66^. Fathollah *et al*.^67^ revealed that helium plasma treatment could activated TGF-β1 cytokines which may lead to enhancement of keratinocyte cell migration and formation of the keratin layer in diabetic rats. The highly expressed TGF-β (Fig. 9) correlated well to the promoted behavior in wound healing stages. Nitric oxide contributed to wound contraction, blood vessel formation and TGF-β expression which corresponds to the improvement from HE and immunohistology. In this research, we could not distinguish what reactive oxygen species actually participated in which process; nevertheless, the level change of antioxidants and the improvement of wound recovery can give us a connection of ROS and wound healing. To show that enhanced wound healing is not only caused by ROS but also NO participant, the NO measurement of plasma-exposed liquid was carried out. The results showed it is also a NO-contained plasma jet and may be involved in the healing process from observation of wound kinetics and histology. Some studies demonstrated statistically significant sterilization on wounds using atmospheric plasma jet with a short time treatment or increase the rate of infected wound closure^68,69^. The application of atmospheric plasma jet could be possibly developed into a new therapy for chronic wound healing.

## Conclusion

In this study, we successfully used an argon-based APPJ in rat models of diabetic wound healing. The wound area ratio of plasma-treated small wounds was greatly reduced compared with that of untreated control wounds. Histology analysis revealed faster re-epithelialization, collagen deposition, less inflammation, and a complete skin structure of the epidermis and dermis layer in the plasma-treated groups compared with the control groups of Type 1 and Type 2 diabetic rats. Moreover, in the IHC results, the new blood vessels of plasma-treated tissues decreased in the middle and late stages of wound healing. The plasma-treated wounds demonstrated more TGF-β expression in the early stage, while they decreased gradually in later days. In the groups with large wounds, the large wound area ratio of Type 1 and Type 2 diabetic rats was reduced.

In addition, the levels of antioxidant in tissue, including SOD, GPx, and CAT, were increased after plasma treatment due to excess ROS from plasma exposure. Moreover, a longer plasma treatment time increased the concentration of H2O2 and the total amount of NO_2_^−^ and NO_3_^−^.
