# Author Correction: Wound Healing in Streptozotocin-Induced Diabetic Rats Using Atmospheric-Pressure Argon Plasma Jet

**DOI:** 10.1038/s41598-018-31449-8

**Published:** 2018-08-29

**Authors:** Kuang-Yao Cheng, Zhi-Hua Lin, Yu-Pin Cheng, Hsien-Yi Chiu, Nai-Lun Yeh, Tung-Kung Wu, Jong-Shinn Wu

**Affiliations:** 1 Department of Mechanical Engineering, National Chiao Tung University, 1001 Ta-Hsueh Road, Hsinchu, 300 Taiwan; 2 Department of Biological Science and Technology, National Chiao Tung University, 75 Bo-Ai Street, Hsinchu, 300 Taiwan; 3 Department of Dermatology, Cathay General Hospital, 280 Renai Road Section 4, Taipei, 106 Taiwan; 4 Department of Dermatology, National Taiwan University Hospital Hsinchu Branch, 25 Jingguo Road Section 1 Lane 442, Hsinchu, 300 Taiwan; 5 Department of Family Medicine, National Taiwan University Hospital Hsinchu Branch, 25 Jingguo Road Section 1 Lane 442, Hsinchu, 300 Taiwan

Correction to: *Scientific Reports* 10.1038/s41598-018-30597-1, published online 15 August 2018

This Article contains a typographical error in the Conclusion section,

“Moreover, a longer plasma treatment time increased the concentration of H2O2 and the total amount of NO_2_^−^ and NO_3_^−^.”

should read:

“Moreover, a longer plasma treatment time increased the concentration of H_2_O_2_ and the total amount of NO_2_^−^ and NO_3_^−^.”

In addition, the Acknowledgements section in this Article is incomplete.

“The authors would like to thank the financial support of the Ministry of Science and Technology of Taiwan (Grant No. MOST-104-2221-E-009-150-MY3) and National Chiao Tung University (Grant No. HCH104-015). We would also like to thank the Pathology Core Laboratory of the National Health Research Institutes (NHRI) of Taiwan and Dr. Cher-Wei Liang of Department of Pathology, Fu Jen Catholic University Hospital and Fu Jen Catholic University College of Medicine of Taiwan for helpful discussion during the research.”

should read:

“The authors would like to thank the financial support of the Ministry of Science and Technology of Taiwan (Grant No. MOST-104-2221-E-009-150-MY3 and MOST-107-2221-E-009-072-MY3) and National Chiao Tung University (Grant No. HCH104-015). We would also like to thank the Pathology Core Laboratory of the National Health Research Institutes (NHRI) of Taiwan and Dr. Cher-Wei Liang of Department of Pathology, Fu Jen Catholic University Hospital and Fu Jen Catholic University College of Medicine of Taiwan for helpful discussion during the research.”

